# Prognostic association of plasma NT-proBNP levels in patients with microvascular angina -A report from the international cohort study by COVADIS-

**DOI:** 10.1016/j.ijcha.2022.101139

**Published:** 2022-10-31

**Authors:** Akira Suda, Jun Takahashi, Maike Schwidder, Peter Ong, Daniel Ang, Colin Berry, Paolo G. Camici, Filippo Crea, Juan Carlos Kaski, Carl Pepine, Ornella Rimoldi, Udo Sechtem, Satoshi Yasuda, John F. Beltrame, C. Noel Bairey Merz, Hiroaki Shimokawa, on behalf of the Coronary Vasomotor Disorders International Study COVADIS Group

**Affiliations:** a Department of Cardiovascular Medicine, Tohoku University Graduate School of Medicine, Sendai, Japan; b Department of Cardiology and Angiology, Robert-Bosch-Krankenhaus, Stuttgart, Germany; c British Heart Foundation Glasgow Cardiovascular Research Centre, Institute of Cardiovascular and Medical Sciences, University of Glasgow, UK; d Vita Salute University and San Raffaele Hospital, Milan, Italy; e Fondazione Policlinico Universitario A. Gemelli IRCCS, Università Cattolica del Sacro Cuore, Rome, Italy; f Cardiovascular and Cell Sciences Res Institute, St George’s, University of London, UK; g Division of Cardiovascular Medicine, University of Florida, College of Medicine, Gainesville, FL, USA; h Institute of Molecular Bioimaging and Physiology, Consiglio Nazionale delle Ricerche, Segrate, Italy; i The Discipline of Medicine, University of Adelaide, Basil Hetzel Institute, Central Adelaide Local Health Network, Adelaide, South Australia, Australia; j Barbra Streisand Women's Heart Center, Smidt Heart Institute, Cedars-Sinai Medical Center, Los Angeles, CA, USA; k International University of Health and Welfare, Narita, Japan

**Keywords:** Microvascular angina, Brain natriuretic peptides, NT-proBNP, Prognosis

## Abstract

Backgroud

The aim of this study was to assess the prognostic association of plasma levels of *N*-terminal prohormone of brain natriuretic peptide (NT-proBNP) with clinical outcomes of patients with microvascular angina (MVA).

**Methods:**

In this international prospective cohort study of MVA by the Coronary Vasomotor Disorders International Study (COVADIS) group, we examined the association between plasma NT-proBNP levels and the incidence of major adverse cardiovascular events (MACE), including cardiovascular death, non-fatal myocardial infarction, non-fatal stroke, and hospitalization due to heart failure or unstable angina.

**Results:**

We examined a total of 226 MVA patients (M/F 66/160, 61.9 ± 10.2 [SD] yrs.) with both plasma NT-proBNP levels and echocardiography data available at the time of enrolment. The median level of NT-proBNP level was 94 pg/ml, while mean left ventricular ejection fraction was 69.2 ± 10.9 % and E/e’ 10.7 ± 5.2. During follow-up period of a median of 365 days (IQR 365–482), 29 MACEs occurred. Receiver-operating characteristics curve analysis identified plasma NT-proBNP level of 78 pg/ml as the optimal cut-off value. Multivariable logistic regression analysis revealed that plasma NT-proBNP level ≥ 78 pg/ml significantly correlated with the incidence of MACE (odds ratio (OR) [95 % confidence interval (CI)] 3.11[1.14–8.49], P = 0.001). Accordingly, Kaplan-Meier survival analysis showed a significantly worse prognosis in the group with NT-proBNP ≥ 78 (log-rank test, P < 0.03). Finally, a significant positive correlation was observed between plasma NT-proBNP levels and E/e’ (R = 0.445, P < 0.0001).

**Conclusions:**

These results indicate that plasma NT-proBNP levels may represent a novel prognostic biomarker for MVA patients.

## Highlights

• Plasma NT-proBNP levels may be a new prognostic biomarker of microvascular angina.

## 1 Introduction

Up to half of patients undergoing diagnostic coronary angiography for typical chest pain have angiographically normal coronary arteries or non-obstructive coronary artery disease (CAD). [1] These patients with signs and symptoms suggestive of ischemic heart disease with no obstructive coronary arteries (INOCA) are increasingly recognized. [2].

In INOCA patients, coronary functional abnormalities could be involved, including epicardial spasm responsible for vasospastic angina (VSA) and/or coronary microvascular dysfunction (CMD) responsible for microvascular angina (MVA). [3] MVA is typically defined as enhanced contraction (microvascular spasm) and/or impaired vasodilatation of coronary microvessels, leading to inadequate increase in blood flow in response to stress with resultant myocardial ischemia. [4], [5], [6] Thus, CMD can cause myocardial ischemia in a sizeable proportion of angina. [7].

Recently, several studies with either invasive or non-invasive techniques demonstrated that patients with MVA have significantly higher rates of cardiovascular events, as compared with non-anginal control populations, indicating the importance of their identification. [8], [9] As the COronary VAsomotor Disorders International Study (COVADIS) group, we have proposed the diagnostic criteria of MVA [10] and demonstrated the clinical characteristics and prognosis of MVA patients in our international prospective cohort study. [11] Briefly, the diagnosis of MVA is based upon symptoms suggestive of myocardial ischemia in the absence of obstructive CAD (<50 % diameter reduction and/or FFR > 0.80) associated with objective evidence of myocardial ischemia and impaired coronary microvascular function defined by one of the following 4 findings, reduced coronary flow reserve (CFR), microvascular spasm, increased microvascular resistance, and coronary “slow flow phenomenon”. [10] Our international MVA cohort study has demonstrated substantial risk of major adverse cardiac events (MACE), especially hospitalization for unstable angina, with hypertension and previous history of CAD being independent clinical predictors of MACE; [11] however, prognostic biomarkers for MVA remain unclear.

We have previously suggested that CMD may play an important role in patients with heart failure with preserved ejection fraction (HFpEF) in the mechanism of ventricular hypertrophy and fibrosis, contributing to diastolic dysfunction and that natriuretic peptides modulates the effect of CMD leading to ventricular hypertrophy and fibrosis. [12] B-type natriuretic peptide (BNP) is a cardiac neurohormone specifically secreted from the ventricles in response to volume expansion and pressure overload. [13] Thus, plasma levels of BNP have been shown to be diagnostic and/or prognostic biomarker in patients with heart failure. [13] On the other hand, inactive *N*-terminal fragment of pro-brain natriuretic peptide (NT-proBNP) has been reported to be a marker of long-term mortality in stable angina patients with obstructive coronary atherosclerosis. [14], [15] However, a possible relationship between plasma NT-proBNP levels and prognosis of MVA patients has not been investigated. Thus, in the present study, we aimed to determine whether plasma NT-pro BNP levels are a novel prognostic biomarker in patients with MVA.

## 2 Methods

### 2.1 International and prospective cohort study on MVA by COVADIS

Details of the international and prospective cohort study of MVA patients have been previously described. [11], [16] Briefly, this cohort study is a multinational, multicenter, multiethnic, prospective, observational, and longitudinal cohort study. We enrolled 686 eligible patients fulfilling the COVADIS diagnostic criteria for MVA as follows; (1) signs and/or symptoms of myocardial ischemia, (2) absence of obstructive CAD, (3) objective evidence of myocardial ischemia, and (4) evidence of impaired coronary microvascular function, as determined by the enrolling site (**Supplemental methods, Supplemental** Table S1). [11].

Patients with obstructive CAD were excluded, which was defined as the presence of any coronary stenosis > 50 % on invasive angiography or computed tomography angiography. Evidence of myocardial ischemia was obtained by rest/stress ECG and/or non-invasive imaging by assessing either myocardial perfusion with single photon emission computed tomography (SPECT), positron emission tomography (PET), cardiac magnetic resonance (CMR), or left ventricular wall motion abnormality with stress echocardiography. [16] Coronary microvascular function was assessed invasively by using coronary functional testing, including measurements of CFR and/or microvascular resistance and/or acetylcholine provocation testing for coronary microvascular spasm. [16].

During the period from July 1, 2015 to December 31, 2018, the participating centers prospectively enrolled patients with MVA. All patients underwent clinical assessments and received usual medical care as determined by attending physicians. Follow-up of each patient was conducted at least once from study entry to the end of December 2019 either by a telephone call or a site visit, depending on the approach considered most practical and effective (**Supplemental methods)**.

The ethics committee of Tohoku University Graduate School of Medicine approved the study protocol (No. 2015-1-188) followed by the ethics committee and/or sponsors at each participating institute, in compliance with the Declaration of Helsinki (UMIN000035177) **(Supplemental methods)**.

### 2.2 Study population

**Fig. 1:**
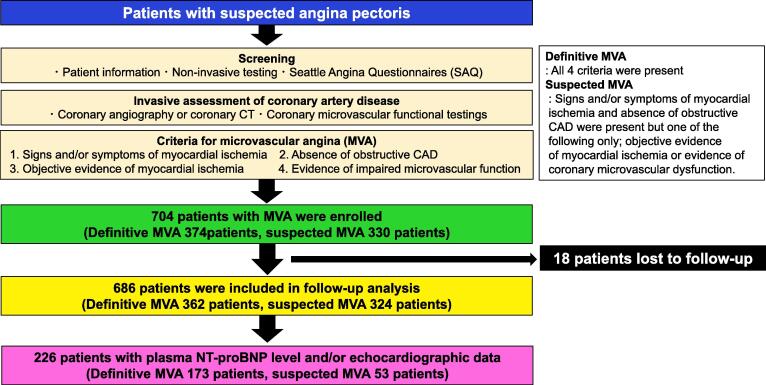
Patient enrollment and follow-up.

Of the 686 patients registered in the COVADIS MVA cohort study, [11] we finally included 226 consecutive patients who had both baseline plasma NT-proBNP levels and echocardiographic data including LV ejection fraction (LVEF) and E/e’ (Fig. 1). At enrollment, we also obtained clinical details, including patient demographic profiles, cardiovascular risk factors, past history of CAD including acute coronary syndrome and stable angina pectoris, non-invasive markers of myocardial ischemia, invasive assessment of microvascular function, initial treatment after diagnosis and assessment of health status by the Seattle Angina Questionnaire (SAQ). [16].

### 2.3 Study endpoints

The primary endpoint was the composite of major cardiovascular events (MACE), including cardiovascular death, non-fatal myocardial infarction (MI), non-fatal stroke, and hospitalization due to heart failure or unstable angina (UA), [11] which were determined by the institutional investigators at each site or an independent clinical event committee. Definition of MI was based on the Third Universal Definition, [17] and that of UA in the presence of ischemic chest pain and hospitalization within 24 h of most recent symptoms, without elevation in cardiac biomarkers but with ischemic ECG changes. [18] Stroke was defined as neurological deficit due to an ischemic or hemorrhagic central nervous system event with residual systems > 24 h after onset or leading to death. [19] For each patient, a MACE was defined as the first occurrence of one of these events during follow-up period. The associations between plasma NT-proBNP levels, echocardiographic data, and MACE were evaluated.

### 2.4 Statistical method

Continuous variables are presented as mean ± SD or medians and interquartile range, depending on the distribution of the data that was tested by Shapiro-Wilk normality test. Categorical variables are presented as counts and percentages. We used the Wilcoxon rank-sum test to compare continuous variables and the Pearson chi-square test to compare categorical variables. Events were analyzed as time from enrolment to first occurrence of any event from the composite endpoint. We used the Kaplan-Meier method to provide survival estimates, which were assessed with a log-rank test. C-statistics, which equal to the area under the Receiver Operating Characteristic curve (AUC), was used to summarize the performance of the predicted probability of the outcomes for discrimination. Additional supportive analyses included time to first occurrence of each component of the composite endpoint individually. Event rate of the composite endpoint and that of each of endpoint are reported separately at 1, 2, and 3 years since enrolment. To examine the association between plasma NT-proBNP levels and incidence of primary endpoint, we used multivariable logistic regression model. A P-value < 0.05 was taken as statistically significant.

## 3 Results

### 3.1 Baseline patient characteristics

We analyzed 226 consecutive patients (M/F 66/160, 61.9 ± 10.2 [SD] yrs.) who had both baseline plasma NT-pro BNP levels and echocardiographic data including LVEF and E/e’. Their clinical characteristics are summarized in Table 1. Baseline patient characteristics were similar between the present study (N = 226) and excluded patients (N = 460) in the prospective cohort study of MVA patients by COVADIS (**Supplemental** Table S2). More than half of patients (71 %) were female and the main ethnic groups were Caucasians (98 %). >50 % had hypertension (62 %) and/or dyslipidemia (81 %), whereas relatively fewer patients had diabetes mellitus (12 %) or were current smokers (17 %). Cardiac function, including LVEF (69.2 ± 10.9) and E/e’ (10.7 ± 5.2), was almost normal and comparable in both sexes (Table 1). However, plasma NT-proBNP levels (median 94 pg/ml [IQR 45–190]) were higher in females than in males (Table 1). Of note, regarding SAQ scores, women tended to have worse scores compared with men in several items, indicating lower QOL in MVA women (Table 1**)**.

**Table 1:** Baseline patient characteristics.

Characteristics	Total cohort (N = 226)	Male (N = 66)	Female (N = 160)	P value
Age (mean, yrs.)	61.9 ± 10.2	61.2 ± 11.3	62.2 ± 9.7	0.61
Race or ethnic group, n (%)				
Caucasian	221 (98)	64 (97)	157 (98)	
Asian	0 (0)	0 (0)	0 (0)	
Hispanic	1 (0.4)	0 (0)	1 (0.6)	
Black	1 (0.4)	1 (1.5)	0 (0)	
Others	3 (1)	1 (1.5)	2 (1.3)	
Body mass index (mean)	27.0 ± 5.2	28.5 ± 4.8	26.2 ± 5.3	0.01
Hypertension, n (%)	140 (62)	47 (71)	93 (58)	0.06
Dyslipidemia, n (%)	183 (81)	56 (85)	127 (79)	0.33
Diabetes mellitus, n (%)	26 (12)	7 (11)	19 (12)	0.78
Current smoking, n (%)	38 (17)	9 (14)	29 (18)	0.40
Previous history of CAD, n (%)	77 (34)	25 (38)	52 (33)	0.44
Previous PCI, n (%)	14 (6)	8 (12)	6 (4)	0.02
LVEF (mean, %)	69.2 ± 10.9	69.4 ± 10.5	69.1 ± 11.1	0.86
E/e’	10.7 ± 5.2	8.9 ± 3.1	11.2 ± 5.6	0.07
NT-pro BNP (pg/ml)	94 (45–190)	50 (23–145)	109 (66–197)	0.0001
Seattle Angina Questionnaire score (median, IQR)				
Physical limitation	64 (39–86)	74 (39–97)	61 (40–81)	0.04
Angina stability	50 (50–75)	50 (50–100)	50 (25–75)	0.06
Angina frequency	70 (50–80)	70 (50–83)	70 (50–80)	0.42
Treatment satisfaction	81 (56–94)	78 (61–94)	81 (56–94)	0.99
Disease perception	42 (25–67)	42 (25–67)	42 (25–67)	0.88
Initial treatment after diagnosis				
Statin, n (%)	193 (85)	59 (89)	134 (84)	0.26
Nitrate, n (%)	132 (58)	35 (53)	97 (61)	0.29
Calcium channel blocker, n (%)	21 (9)	7 (11)	14 (9)	0.67
Beta blocker, n (%)	104 (46)	26 (39)	78 (49)	0.20
Angiotensin-converting enzyme inhibitor, n (%)	62 (27)	19 (29)	43 (27)	0.77
Angiotensin Ⅱ receptor blocker, n (%)	65 (29)	23 (35)	42 (26)	0.20

CAD, coronary artery disease; IQR, interquartile range; NT-pro BNP, *N*-terminal prohormone of brain natriuretic peptide; PCI, percutaneous coronary intervention; SAQ, Seattle angina questionnaire.

### 3.2 Clinical outcomes and plasma NT-proBNP levels

**Fig. 2:**
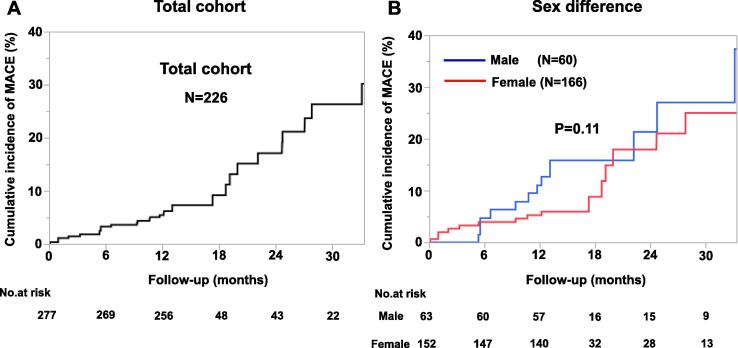
Kaplan-Meier curves for MACE (A) Total cohort (n = 226). (B) Sex difference.

**Fig. 3:**
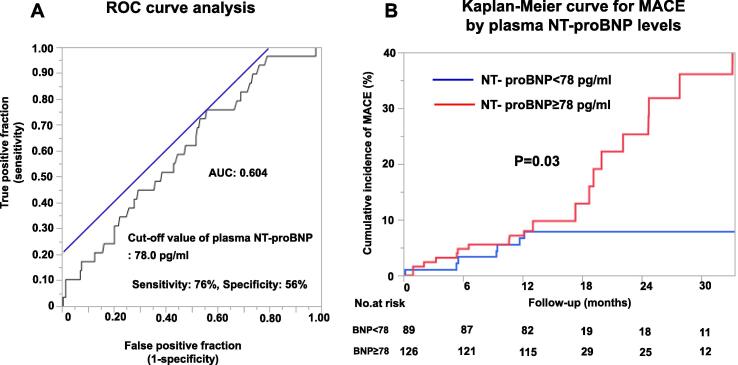
Correlation between plasma NT-proBNP levels and incidence of MACE. (a) ROC curve of plasma NT-proBNP levels for MACE. In order to obtain the cut-off value of NT-proBNP, we employed the Youden’s index method where the summed sensitivity and specificity became the largest. The blue line represents the line that passes the point where the summed sensitivity and specificity became the largest with 45 degree tilt. (B) Kaplan-Meier curve for MACE by plasma NT-proBNP level of 78 pg/ml. (For interpretation of the references to colour in this figure legend, the reader is referred to the web version of this article.)

During the median follow-up period of 365 days (IQR 365, 482 days), 29 MACEs were recorded, including hospitalization for unstable angina (N = 22), cardiovascular death (N = 3), non-fatal myocardial infarction (N = 3), and hospitalization for heart failure (N = 1). The annual incidence of composite MACE primary endpoint was 7.9 % per patient year (Fig. 2**A**) and there was no significant sex difference (male 12.7 % vs female 5.9 % per patient year, P = 0.11) (Fig. 2**B**). Based on the receiver-operating characteristics (ROC) curve analysis, the optimal cutoff value of plasma NT-proBNP level for developing MACEs was 78.0 pg/ml, and the area under the ROC curve was 0.604 (Fig. 3**A**). With this value, the sensitivity and specificity for predicting MACE were 76 % and 56 %, respectively (Fig. 3**A**). Multivariable logistic regression analysis showed that plasma NT-proBNP level ≥ 78 pg/ml significantly correlated with the incidence of MACE (odds ratio (OR) [95 % confidence interval (CI)] 3.11[1.14–8.49], P = 0.03) **(**Table 2). Importantly, when we divided the patients into the 2 groups by the cut-off value of plasma NT-proBNP level, the Kaplan-Meier survival analysis showed a significantly worse prognosis in the group with NT-proBNP ≥ 78 compared with that with NT-proBNP < 78 (log lank, P = 0.03) (Fig. 3**B)**.

**Table 2:** Prognostic factors for MACE in patients with MVA (logistic regression model).

	Univariable analysis			Multivariable analysis		
	OR	95 % CI	P value	OR	95 % CI	P value
Female sex	0.453	0.204 – 1.018	0.06	0.356	0.144 – 0.879	0.03
Hypertension	2.104	0.897 – 5.533	0.09			
Dyslipidemia	2.208	0.729 – 9.581	0.17			
Current smoking	0.534	0.123 – 1.628	0.29			
Previous history of CAD	2.744	1.246 – 6.151	0.01			
NT pro-BNP ≥ 78 pg/ml	2.486	1.061 – 6.531	0.04	3.113	1.145 – 8.491	0.03

CAD, coronary artery disease; CI, confidence interval; LVEF, left ventricular ejection fraction; MACE, major adverse cardiac event; MVA, microvascular angina; NT-pro BNP, *N*-terminal prohormone of brain natriuretic peptide; OR, odds ratio.

### 3.3 Correlation between plasma NT-proBNP levels and cardiac functions

**Fig. 4:**
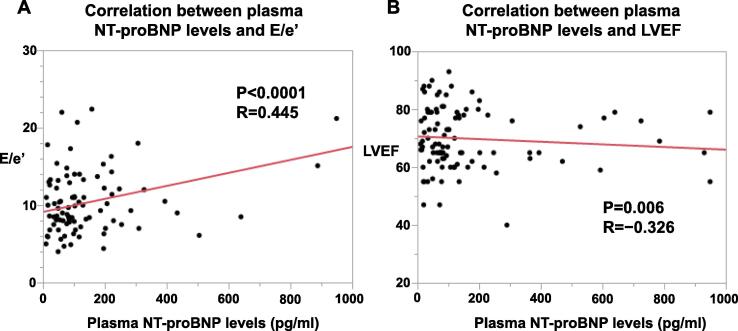
Correlation between plasma NT-proBNP levels and cardiac functions. (A) Correlation between plasma NT-proBNP levels and E/e’ (diastolic function). (B) Correlation between plasma NT-proBNP levels and LVEF (systolic function).

In the present study, we found a significant positive correlation between plasma NT-proBNP levels and E/e’ (R = 0.445, P < 0.0001) (Fig. 4**A**). In addition, LV systolic function (LVEF) was also weakly but significantly correlated with plasma NT-proBNP levels in MVA patients (R =  − 0.326, P = 0.006) (Fig. 4**B**).

## 4 Discussion

The major findings of the present study in patients with MVA demonstrates that plasma NT-proBNP levels; (1) were higher in females than in males, (2) correlated with E/e’ and LVEF in echocardiography, and (3) significantly correlated with the occurrence of MACE, with 78.0 pg/ml being the best cut-off value. To the best of our knowledge, this is the first study that demonstrates the prognostic value of plasma NT-proBNP levels in patients with MVA.

### 4.1 Clinical settings in MVA and HFpEF patients

Coronary microvascular dysfunction (CMD) can occur in many clinical settings and can be triggered by multiple pathogenetic mechanisms. [5] Indeed, CMD is attributable to disruption of normal coronary physiology, which may subsequently impair the capacity of myocardial blood flow to meet myocardial oxygen demand. [4] Recently, some studies reported that CMD can be demonstrated not only in MVA patients but also in patients with HFpEF. [20], [21] The diagnosis of HFpEF is based on the following; (1) symptoms with or without signs of heart failure, (2) normal or only slightly reduced LVEF, (3) elevated levels of natriuretic peptides, and (4) relevant structural heart disease and/or diastolic dysfunction. [12] In addition, angina or angina-like symptoms are present in about 50 % of patients with HFpEF. [12] In the present study, predominant symptoms were chest pain or chest discomfort and systolic function of enrolled patients was within the normal range. Moreover, their plasma NT-proBNP levels were elevated, especially in females (Table1). These findings endorse the hypothesis that similar clinical conditions could co-exist between MVA and HFpEF. [12].

### 4.2 Prognostic impact of plasma NT-proBNP levels

Although it has been suggested that the prognosis of MVA patients is good, [22] our international prospective cohort study and other follow-up studies have demonstrated considerable risk of MACE in these patients. [8], [9], [11] In the present study, the incidence of the composite MACE endpoint in the overall cohort (7.9 % per patient year, Fig. 2**A**) was comparable to that reported by Pepine et al. [23] Furthermore, for the first time, we were able to demonstrate the prognostic impact of plasma NT-proBNP levels with the best cut-off value being 78.0 pg/ml in patients with MVA (Fig. 3**A**). The cut-off value of plasma NT-proBNP levels was considerably lower than previously reported for the diagnosis of heart failure, [13] with a level > 78.0 pg/ml associated with increased incidence of MACE (Table 2). It was previously reported from the Framingham Heart Study that NT-proBNP levels in healthy individuals were substantially higher in women than in men at every age, and levels increased with increasing age for both sexes. [24] Importantly, when we categorized the patients into the 2 groups by the cut-off plasma NT-proBNP level, those with NT-proBNP ≥ 78 pg/ml had a worse prognosis compared with those with NT-proBNP < 78 pg/ml (Fig. 3**B**).

### 4.3 Sex differences in MVA patients

Previous studies have found that women were more likely to have angina without obstructive CAD, yet have a comparable risk of cardiovascular events as compared with men. [[2], [25]] In the present study, there was no significant sex difference in the incidence of cardiovascular events (Fig. 2**B**). Additionally, as demonstrated in Table 1**,** women tended to have lower SAQ scores and higher plasma NT-proBNP levels than men, despite the fact that there were no significant sex differences in their cardiac function. Recently, the CorMicA randomized, controlled trial of stratified medicine reported improvements in anginal symptoms and QOL in patients with CMD in general, but without sex-related differences. [8] However, female hormones are involved in sex differences in perception of chest symptoms in females not only in MVA patients but also in HFpEF patients. [12] These findings raise an important issue that elevation of NT-proBNP may relate to lower QOL and future cardiovascular events in female patients with MVA.

### 4.4 Correlation between plasma NT-proBNP levels and cardiac functions

In the present study, a significant correlation was noted between plasma NT-proBNP levels and E/e’ (diastolic function) rather than LVEF (systolic function), suggesting the presence of cardiac diastolic dysfunction in patients with MVA (Fig. 4). This finding also suggests that a common underlying mechanism of cardiac diastolic dysfunction exists in both MVA and HFpEF. [12].

### 4.5 Study limitations

Although our study has several strengths (the first international study with multiple ethnicities and countries, large sample size, use of consensus diagnostic criteria for MVA, and high follow-up rate, etc.), several limitations should be considered. First, the present study was observational without a reference group. Second, the relatively small number of MACE during follow-up limits the statistical power of the present study and might have led to data overfitting. Third, most MACE (76 %) reflected hospitalization for unstable angina. However, the prevalence of hospitalization for unstable angina to total MACE was comparable with the previous reports. [[9], [26]] Fourth, we excluded patients with obstructive CAD by conventional angiography or coronary computed tomography and have no data regarding functional relevance of coronary artery stenoses evaluated by physiological indices. Fifth, since we aimed to examine the prognostic significance of plasma NT-proBNP levels, we examined the association between the NT-proBNP levels and MACE, but not other laboratory parameters. We would like to address this point in future studies. Finally, we have no data regarding changes in or adherence to medical therapy, or symptoms and/or QOL (e.g. SAQ) during follow-up. These issues remain to be examined in future studies.

## 5 Conclusions

In the present study, we were able to demonstrate that in patients with MVA, plasma levels of NT-proBNP could be a novel prognostic biomarker, suggesting an involvement of common underlying mechanisms as in HFpEF.

## 6 Take-home message

We demonstrate that in patients with microvascular angina, plasma levels of NT-proBNP could be a novel prognostic biomarker, suggesting an involvement of common underlying mechanisms as in heart failure with preserved ejection fraction.

**Sources of funding:** The Japan Heart Foundation.

**Registration number:** UMIN000035177.

## 7 Author statement

F.C. reports speaker fees from AstraZeneca, Amgen and Servier and institutional agreements between his employer, the Catholic University, and Biotronik, Boheringer Ingelheim. C.N.B.M. reports lecturer fees from Abbott Diagnostics, board director fees from iRhythm, consulting fees from Caladrius, and advisory board fees from Bayer. C.B. declares institutional agreements between his employer, the University of Glasgow, and AbbottVascular, AstraZeneca, Boehringer Ingelheim, Coroventis, DalCor, GSK, HeartFlow, Novartis, and Philips. P.G.C. reports speaking honoraria from Servier and Abbott. P.O. reports personal fees from Bayer Healthcare, Pfizer and Philips/Volcano. U.S. reports speaker and consulting fees from Amgen, Bristol-Myers Squibb, Boehringer- Ingelheim, Abbott, Servier, Astra-Zeneca, Bayer, and Pfizer. T.F. has acted as a speaker for Abbott Vascular, Boehringer Ingelheim and Novartis. None of the declared interests regard the submitted work. All other authors have nothing to disclose.

## CRediT authorship contribution statement

**Akira Suda:** Software, Investigation, Formal analysis. **Jun Takahashi:** Investigation, Formal analysis. **Maike Schwidder:** Investigation. **Peter Ong:** Investigation. **Daniel Ang:** Investigation. **Colin Berry:** Investigation. **Paolo G. Camici:** Investigation. **Filippo Crea:** Investigation. **Juan Carlos Kaski:** Investigation. **Carl Pepine:** Investigation. **Ornella Rimoldi:** Investigation. **Udo Sechtem:** Investigation. **Satoshi Yasuda:** Investigation. **John F. Beltrame:** Investigation, Supervision. **C. Noel Bairey Merz:** Investigation, Supervision. **Hiroaki Shimokawa:** Conceptualization, Methodology, Writing – original draft, Funding acquisition.

## Declaration of Competing Interest

The authors declare that they have no known competing financial interests or personal relationships that could have appeared to influence the work reported in this paper.
