# Supplementary material for: Prognostic association of plasma NT-proBNP levels in patients with microvascular angina -A report from the international cohort study by COVADIS-
Source: Int J Cardiol Heart Vasc. 2022 Oct 31;43:101139. doi: 10.1016/j.ijcha.2022.101139 (PMC9626381; doi:10.1016/j.ijcha.2022.101139)
Supplement: Supplementary data 1 [file mmc1.docx]

**Prognostic association of plasma level of NT-proBNP in patients with microvascular angina**

**-A report from the international cohort study by COVADIS-**

**Supplementary materials**

**Supplementary methods**

**Definitions**

We diagnosed patients as having microvascular angina by using the COVADIS diagnostic criteria as follows; (1) signs and/or symptoms of myocardial ischemia, (2) absence of obstructive CAD, (3) objective evidence of myocardial ischemia, and (4) evidence of impaired coronary microvascular function, as determined by the clinical site (**Table S1**).^1^

**Data collection**

All patients who met the eligibility criteria determined at the participating site were registered following the site ethical review board approval. Data collection was performed through the use of the electronic case report form established by the Japanese Coronary Spasm Association.^2^ The investigators at each site registered information on demographics, relevant medical history, cardiovascular risk factors, quality of life (e.g. Seattle Angina Questionnaire, SAQ),^3^ diagnostic approaches for myocardial ischemia, anatomical and/or functional status of epicardial coronary arteries and coronary microcirculation, and medications. Follow-up of each patient was conducted at least once from study entry to the end of December 2019 either by a telephone call or personal visit, depending on the approach considered most practical and effective.

**Study variables**

Study variables obtained at enrolment included patient demographics (sex, age, height, and weight), cardiovascular risk factors (hypertension, hyperlipidemia, diabetes mellitus, smoking, and menopause), past and family history of coronary artery disease (CAD) including acute coronary syndrome and stable angina pectoris, type of angina episodes (effort, rest, or mixed), circadian distribution of angina attacks, ECG leads of ST-segment elevation or depression at rest, arrhythmias during spontaneous attack, use of non-invasive diagnostic modalities for myocardial ischemia (SPECT, PET, CMR, stress echocardiography or electrocardiography), information regarding interventional diagnostic procedures for assessment of coronary vasodilatation (e.g. coronary flow reserve, index of microcirculatory resistance, hyperemic microvascular resistance) or assessment for propensity to coronary vasoconstriction (e.g. spasm provocation testing), medications (calcium channel blocker, nitrate, statin, ACE-I, ARB, and beta-blocker), patient-reported angina status assessed by the SAQ. During the follow-up period, clinical outcomes (cardiovascular death, non-fatal MI, non-fatal stroke, hospitalization due to heart failure, and UA) were collected.

**Echocardiography**

Doppler echocardiography was performed in each participating site. Peak Doppler velocities of early and late diastolic flow, deceleration time and E/A ratio were measured. Additionally, tissue Doppler imaging of mitral annulus was obtained from the apical 4-chamber view. E/e’ ratio was calculated by using the early diastolic flow velocity (e’) at the lateral and medial mitral annulus.^4^

**Measurement of NT-proBNP level**

Plasma NT-pro-BNP level was measured at each participating site by using immunoassay as previously reported.^5^  The normal range for plasma NT-proBNP levels was defined as lower than 125 pg/ml for adults younger than 75 years and lower than 450 pg/ml for adults 75 years or older.^6^

**Ethics approval**

The present study was performed in accordance with ethical principles that are consistent with the Declaration of Helsinki, International Conference on Harmonization of Good Clinical Practice guidelines, and the applicable legislation on non-interventional studies. The final protocol was approved by the site ethics committee. An investigator at each site ensured that the patient was given full and adequate oral and written information in the local language about the nature, purpose, possible risk, and benefit of the present study.

**Study organization**

The Coronary Vasomotor Disorder International Study (COVADIS) group was established in 2012 to define the nomenclature and stimulate interest into coronary vasomotor disorders. The COVADIS Steering Committee served as the principal investigators for the COVADIS Microvascular Angina Registry, including the Steering Committee co-chairs and the data coordinating center (DCC). The Steering Committee members are as follows; John Beltrame (COVADIS co-chair, Australia), Colin Berry (PI, United Kingdom), Paolo Camici (PI, Italy), Filippo Crea (PI, Italy), Juan Carlos Kaski (PI, United Kingdom), C. Noel Bairey Merz (COVADIS co-chair, USA), Peter Ong (PI, Germany), Carl J Pepine (PI, USA), Udo Sechtem (PI, Germany), and Hiroaki Shimokawa (Study Chair, DCC, Japan).

**Supplementary references**

1. Ong P, Camici PG, Beltrame JF, Crea F, Shimokawa H, Sechtem U, Kaski JC, Merz

CNB. International standardization of diagnostic criteria for microvascular angina. *Int*

*J Cardiol* 2018;**250**:16–20.

2. Suda A, Takahashi J, Beltrame JF, Berry C, Camici PG, Crea F, Escaned J, Ford T, Kaski

JC, Kiyooka T, Metha PK, Ong P, Ozaki Y, Pepine C, Rimoldi O, Safdar B, Sechtem U,

Tsujita K, Yii E, Merz CNB, Shimokawa H. International prospective cohort study of

microvascular angia -Ratinale and design. *Int J Cardiol Heart Vasc*;2020;**31**:100630

Doi:10.1016/j.ijcha.2020.100630.

3. Spertus JA, Winder JA, Dewhurst TA, Deyo RA, Prodzinski J, Mcdonell M, Fihn SD.

Development and evaluation of the Seattle Angina Questionnaire: A new functional

status measure for coronary artery disease. *J Am Coll Cardiol* 2015;**25**:333–341.

4. Ommen SR, Nishimura RA, Appleton CP, Miller FA, Oh JK, Redfield MM, Tajik AJ.

Clinical utility of doppler echocardiography and tissue doppler imaging in the estimation

of left ventricular filling pressures. *Circulation* 2000;102:1788-1794

5. Kragelund C, Gronning B, Kober L, Hildebrandt P, Steffensen R. N-terminal

pro-B-type natriuretic peptide and long-term mortality in stable coronary heart

disease. *N Engl J Med* 2005;352:666-675

6. Corteville DCM, Domingo KB, Wu AHB, Ali S, Schiller NB, Whooley MA. N-

terminal pro-B-type natriuretic peptide as a diagnostic test for ventricular dysfunction in

patients with coronary disease. *Arch Intern Med* 2007;167:483-489

**Supplementary tables**

**Supplementary table 1. Criteria for microvascular angina (MVA) by COVADIS (Ref. 9)**

| **1. Symptoms of myocardial ischemia** |
| --- |
| a. Effort and/or rest angina |
| b. Angina equivalents (i.e. shortness of breath) |
| **2. Absence of obstructive coronary artery disease** (< 50% diameter reduction or FFR > 0.80) by |
| a. Coronary CTA |
| b. Invasive coronary angiography |
| **3. Objective evidence of myocardial ischemia** |
| a. Ischemic ECG changes during an episode of chest pain |
| b. Stress-induced chest pain and/or ischemic ECG changes in the presence or absence of transient/reversible |
| abnormal myocardial perfusion and/or wall motion abnormality |
| **4. Evidence of impaired coronary microvascular function** |
| a. Impaired coronary flow reserve (cut-off values depending on methodology use between < 2.0 and < 2.5) |
| b. Coronary microvascular spasm, defined as reproduction of symptoms, ischemic ECG changes but no epicardial |
| spasm during acetylcholine provocation test |
| c. Abnormal coronary microvascular resistance indices (e.g. IMR > 25) |
| d. Coronary slow flow phenomenon, defined as TIMI frame count > 25 |
| **Definitive MVA**: all four criteria are present for a diagnosis of microvascular angina. |
| **Suspected MVA**: symptoms of ischemia are present with no obstructive coronary artery disease but only objective |
| evidence of myocardial ischemia, or evidence of impaired coronary microvascular function alone. |

CTA, computed tomographic angiography; ECG, electrocardiogram; FFR, fractional flow reserve; IMR, index of microcirculatory resistance;

TIMI, thrombolysis in myocardial infarction.

**Supplementary table 2. Comparison of baseline clinical characteristics between patients included in and those excluded from the present study in the prospective cohort study of MVA patients by COVADIS**

| **Characteristics** | **Present study (N=226)** | **Excluded patients (N=460)** | **P value** |
| --- | --- | --- | --- |
| Age (mean, yrs.) | 61.9±10.2 | 60.6±12.5 | 0.14 |
| Female, n (%) | 160 (71) | 276 (60) | 0.006 |
| Body mass index (mean) | 27.0±5.2 | 25.9±6.0 | 0.01 |
| Hypertension, n (%) | 140 (62) | 220 (48) | 0.0005 |
| Dyslipidemia, n (%) | 183 (81) | 179 (39) | <0.0001 |
| Diabetes mellitus, n (%) | 26 (12) | 40 (19) | 0.008 |
| Current smoking, n (%) | 38 (17) | 69 (15) | 0.63 |
| Previous history of CAD, n (%) | 77 (34) | 147 (34) | 0.96 |
| Previous PCI, n (%) | 14 (6) | 50 (11) | 0.04 |
| LVEF (mean, %) | 69.2±10.9 | 64.1±9.7 | <0.0001 |
| SAQ score (median, IQR) |  |  |  |
| Physical limitation | 64 (39-86) | 81 (61-94) | <0.0001 |
| Angina stability | 50 (50-75) | 50 (25-75) | 0.48 |
| Angina frequency | 70 (50-80) | 70 (60-90) | 0.20 |
| Treatment satisfaction | 81 (56-94) | 75 (63-88) | 0.44 |
| Disease perception | 42 (25-67) | 50 (33-58) | 0.60 |
| Initial treatment after diagnosis |  |  |  |
| Statin, n (%) | 193 (85) | 234 (51) | <0.0001 |
| Nitrate, n (%) | 132 (58) | 161 (35) | <0.0001 |
| Calcium channel blocker, n (%) | 21 (9) | 227 (49) | <0.0001 |
| Beta blocker, n (%) | 104 (46) | 142 (31) | 0.0002 |
| Angiotensin-converting enzyme inhibitor, n (%) | 62 (27) | 104 (23) | 0.18 |
| Angiotensin Ⅱ receptor blocker, n (%) | 65 (29) | 55 (12) | <0.0001 |

CAD, coronary artery disease; LVEF, left ventricular ejection fraction; magnetic resonance imaging; PCI, percutaneous coronary intervention, SAQ, Seattle angina questionnaire
